# Diagnostic performance of a point-of-care molecular system for the detection of SARS-CoV-2 in Peru

**DOI:** 10.17843/rpmesp.2024.411.13046

**Published:** 2024-03-26

**Authors:** Zully M. Puyén, Roger Araujo-Castillo, Jorge Giraldo, Karina Gutierrez, Nancy Rojas-Serrano

**Affiliations:** 1 Instituto Nacional de Salud, Lima, Peru. Instituto Nacional de Salud Lima Peru; 2 School of Medicine, Universidad Peruana de Ciencias Aplicadas, Peru, Lima, Peru. Universidad Peruana de Ciencias Aplicadas School of Medicine Universidad Peruana de Ciencias Aplicadas Lima Peru

**Keywords:** SARS-CoV-2, COVID-19, Molecular Diagnostic Techniques, PCR, Sensitivity and Specificity, Peru

## Abstract

The present study assessed the diagnostic performance of the Xpert®Xpress SARS-CoV-2 test in comparison with the Charité protocol real-time RT PCR for the detection of SARS-CoV-2 in Peruvian patients. This was a diagnostic test study that included 100 nasal and pharyngeal swab samples. We obtained an overall concordance of 98.70% (95%CI: 92.98-99.97), with a kappa coefficient of 0.97 (95%CI: 0.86-1.00) and sensitivity and relative specificity rates of 100% and 96.15%, respectively. Additionally, the percentage of the area under the ROC curve was 98.08% in both cases, and an analytical specificity rate of 100% was obtained for the different respiratory viruses evaluated. In conclusion, the Xpert®Xpress SARS-CoV-2 test, by using nasal and pharyngeal swab samples, was highly sensitive and specific, and the kappa coefficient showed an excellent correlation when compared to the reference test.

## INTRODUCTION

In 2019, the first cases of coronavirus disease (COVID-19) were reported in Wuhan province, China. Subsequently, SARS-CoV-2 virus was identified as the causative agent ^1^, and at the end of February 2020 it was declared a pandemic by the World Health Organization (WHO) ^2^. To control this pandemic, one of the key points was the development and implementation of technologies for the detection of the virus causing the disease, which led to the development of molecular tests that aided in the rapid and timely detection of SARS-CoV-2 ^3^. Different platforms were designed with the aim of finding an alternative that was fast, easy to implement, and efficient. However, most of them require complex infrastructure and highly trained personnel, which in many cases limits the decentralization of molecular testing in Peru.

The GeneXpert platform is a fully automated closed system based on real-time PCR, which integrates in a single system and automates all the procedures of sample preparation, nucleic acid extraction and amplification, as well as target sequence detection ^4^^,^^5^. This system requires the use of disposable cartridges that include the necessary controls to validate the sample run, as well as the detection of target genes. In Peru, by the end of 2020, 38 laboratories had been implemented with this platform by the National Institute of Health (INS) for use in the detection of tuberculosis and/or HIV viral load ^6^^,^^7^. For this reason, one of the intervention strategies was the implementation of the Xpert Xpress SARS-CoV-2 cartridge ^4^, which was developed for the molecular detection of the virus causing COVID-19.

The Xpert Xpress SARS-CoV-2 cartridge specifically detects two very important target genes, namely “N2” (protein nucleocapsid) and “E” (protein envelope), increasing the sensitivity of the test for the detection of SARS-CoV-2 ^8^^,^^9^. The performance obtained for this test in terms of concordance was 100% in agreement with the assay performed using a quantified reference material of virus particles. Likewise, the analytical limit of detection (LoD) was established, which, according to the manufacturer, was 0.02 PFU/ml ^4^.

In view of the above, this study aimed to evaluate the diagnostic performance of the real-time automated nucleic acid amplification test (Xpert®Xpress SARS-CoV-2) in comparison with real-time RT PCR - Charité protocol ^3^, for the qualitative molecular detection of SARS-CoV-2 (COVID-19), from nasal and pharyngeal swab samples contained in universal viral transport medium from Peruvian patients.

**KEY MESSAGES**

Motivation for the study: To describe and evaluate a closed molecular platform, easy to use and of importance in Peru for the management of diseases of public health priority, now implemented for the detection of SARS-CoV-2.

Main findings: Highly sensitive and specific molecular test, with excellent correlation compared to the reference test for detecting SARS-CoV-2.

Implications: Can be used in point-of-care laboratories for rapid molecular detection of different infectious agents, including SARS-CoV-2. Little expertise and minimal infrastructure are required to implement it.

## THE STUDY

### Design and samples

This was an observational, cross-sectional study designed as a diagnostic test evaluation, which was carried out using nasal and pharyngeal swab samples contained in universal transport media collected in 2020 from Peruvian patients and stored in the INS sample bank. These previously tested positive or negative by real-time RT-PCR for SARS-CoV-2.

The Epidat 4.2 program was used to calculate the sample size. Considering a confidence level of 95%, that the RT-PCR classified 50% of samples as positive (50 positive and 50 negative) ^10^, and assuming that the automated amplification test classified a similar number as positive; then a sample size of 100 samples allows finding a Cohen’s kappa coefficient of 0.90 with a precision of +/- 0.085; a lower precision would have meant requiring less sample. The expected kappa coefficient corresponded to a very good correlation, close to the 0.98 reported by Zheng *et al*. ^11^. Finally, the selected samples were previously identified as positive (n=52) and negative (n=50) for SARS-CoV-2 by real-time RT-PCR. Among these 50 negative samples, 25 were positive for other viruses in order to evaluate cross-reactivity.

### RT-PCR platforms and instruments

*Real-time RT-PCR*

RNA extraction was performed according to the manufacturer’s specifications with the QIAamp Viral RNA Mini kit either manually or using the QIACUBE® automated kit. For SARS-CoV-2 detection, the extract was subjected to real-time RT PCR-Charité protocol ^3^ and real-time RT-PCR using primers and probes specific for SARS-CoV-2 RdRP and human GAPDH genes ^12^. Multiplex real-time RT-PCR in TaqMan system (qRT-PCR_Multiplex) was used for the detection of influenza A and influenza B viruses ^(^^13^. Finally, for the detection of respiratory syncytial virus, human rhinovirus and metapneumovirus, we used a multiplex RT-PCR for other respiratory viruses designed at the INS of Peru. 

*Automated Real-Time Nucleic Acid Amplification Assay*

Xpert® Xpress SARS-CoV-2 was performed according to the manufacturer’s instructions ^4^. Briefly, 300 µL of sample was added to the cartridge, which was closed and then placed in a GeneXpert IV kit to perform the test. The cartridges include two internal controls (SPC -sample processing control- and PCC -probe check control-) that ensure the correct functioning of the test ^5^. SARS-CoV-2 detection is based on the identification of two genes: “N2” (protein nucleocapsid) and “E” (protein envelope), which are used for the interpretation of results. The results are automatically generated by the GeneXpertTM DX Software version 6.2. Results are reported qualitatively, and the analyzed samples are considered positive when both N2 and E genes are detected or when only N2 is detected. When only the E gene was detected, the manufacturer’s recommended interpretation was a presumptive positive result; however, it was considered positive during the pandemic. This is because, at the beginning of the pandemic, there were molecular detection tests designed for betacoronaviruses, and SARS-CoV-2 was the most prevalent virus of this genus during the pandemic, so at that time the implementation of confirmation by detection of a single genetic marker was recommended, taking into account that the curves, as well as other quality assurance parameters, were optimal. PCR of the E gene showed better sensitivity, so it was recommended to prioritize the E gene as the selected marker ^14^. However, later tests based on protein S began to be performed, which were more specific, and that is why, this type of markers were additionally included for detection for the later tests.

### Statistical analysis

Stata v15.1 (Stata Corporation, College Station, Texas, USA) was used for data analysis. Qualitative variables were reported as absolute and relative frequencies. Numerical variables were described with median and interquartile range (IQR), because the small sample size does not guarantee a normal distribution; and with range of minimum and maximum values. Concordance, relative diagnostic performance, Cohen’s kappa coefficient, with 95% confidence intervals were analyzed. Likewise, relative sensitivity (percentage of positive concordance) and relative specificity (percentage of negative concordance) were obtained using real-time RT-PCR as an imperfect reference standard, since the ideal reference standard is virus isolation in cell culture from different patient samples; however, due to the complexity of the procedure this is not usually used, so real-time RT-PCR was used as a surrogate. The area under the ROC (Receiver operating characteristic) curve was also calculated as a percentage with its respective confidence interval.

On the other hand, analytical specificity was obtained using only RT-PCR negative tests for SARS-CoV-2 but positive for other respiratory viruses. All evaluations were performed at a significance level of 0.05. The different obtained parameters were compared by evaluating their cross-confidence intervals. If their 95% confidence intervals did not cross, they were considered statistically different.

### Ethical aspects

This study was approved by the INS ethics committee, with document No. RD: 176-2020-OGITT/INS.

## RESULTS

We included 102 samples, 77 from COVID-19 suspect patients, collected between April and September 2020; and 25 from pre-pandemic samples stored at -80 °C in the INS sample bank. The former were used to calculate the relative performance of automated amplification, and the latter to evaluate the analytical specificity of the test. Of the first group, 52 were positive and 25 were negative for SARS-CoV-2 RT-PCR.

Of the samples, 55.8% (43/77) came from males. Women had a median age of 40.5 years (IQR: 34-49, minimum-maximum: 0-60), and men, 47 years (IQR: 38-56, minimum-maximum: 3-85). Out of the samples from pre-pandemic patients, 44.0% were women (11/25). There was only one discrepancy when comparing both tests in patients with clinical suspicion of COVID-19, with one sample being negative by RT-PCR and positive by automated amplification. This yielded an overall concordance of 98.70% (95%CI: 92.98-99.97), with a kappa coefficient of 0.97 (95%CI: 0.86-1.00), corresponding to an excellent correlation. The numbers were very similar when two “presumptive positives” in the automated amplification test, initially considered as positive, were excluded (Table 1 and 2).

**Table 1:** Comparison of results obtained by automated amplification for the detection of SARS-CoV-2, with the real-time RT-PCR test.

Positivity criterion	Automated amplification result	Results of Real-time RT-PCR		
		Positive	Negative	Total
Suspected positives are considered positive	Positive	51	1	52
	Negative	0	25	25
	Total	51	26	77
Suspected positives are excluded from the test	Positive	49	1	50
	Negative	0	25	25
	Total	49	26	75

**Table 2:** Diagnostic performance of automated amplification for the detection of SARS-CoV-2 compared to real-time RT-PCR.

		SARS-COV-2 detection		
		N ^a^	Result (%)	95%CI
Considering presumptive positive results				
	Overall concordance	76/77	98.70	92.98-99.97
	Cohen’s kappa coefficient		0.97	0.86-1.00
	Area under ROC curve		98.08	94.31-100.00
	Relative sensitivity	51/51	100.00	93.02-100.00
	Relative specificity	25/26	96.15	80.36-99.90
Excluding presumptive positive results				
	Overall concordance	74/75	98.67	92.79-99.97
	Cohen’s kappa coefficient		0.97	0.85-1.00
	Area under ROC curve		98.08	94.31-100.00
	Relative sensitivity	49/49	100.00	92.75-100.00
	Relative specificity	25/26	96.15	80.36-99.90

a Data evaluated for the specific analysis; 95%CI: 95% confidence interval.

ROC: Receiver operating characteristic

The relative performance evaluation with respect to real-time RT-PCR for SARS-CoV-2 showed a relative sensitivity of 100%, both when all samples were included and when the two “presumptive positives” in the amplification test were excluded. The relative specificity was 96.15% and the % area under the ROC curve was 98.08% in both cases (Table 1 and 2).

In addition, Table 3 presents the medians with their IQRs for the RdRP, ORF1a, E, and N genes from the real-time RT-PCR, and for the N2, and E genes from the automated amplification platform used. Overall, we found a wide range of values for all the evaluated genes.

**Table 3:** Median Ct values of positive tests to the different genes used by real-time RT-PCR and automated amplification for SARS-CoV-2 detection.

Platform	Gen	N	Median	IQR	Min-max
Real-time RT-PCR	RDRP	32	22.83	17.71-26.23	10.52-30.55
	ORF	18	21.05	16.82-25.40	13.25-31.81
	GEN_E	26	28.05	23.97-31.97	12.51-38.28
	GEN_N	25	21.62	18.44-27.80	14.67-34.89
Automated amplification	N2_XPERT	50	27.45	21.40-30.70	12.00-39.80
	E_XPERT	49	26.10	18.50-28.50	11.00-44.60

IQR: interquartile range; Min-max: minimum value and maximum value

Finally, the analytical specificity was 100% for all included respiratory viruses, including respiratory syncytial virus (5/5), human rhinovirus (6/6), metapneumovirus (5/5), influenza A virus (5/5), and influenza B virus (5/5) (Table 4).

**Table 4:** Analytical specificity of automated amplification for SARS-CoV-2 detection, relative to real-time RT-PCR, for different respiratory viruses.

	SARS-CoV-2				Analytical specificity
	Real-time RT-PCR		Automated amplification		
	Positive	Negative	Positive	Negative	
Respiratory Syncytial Virus (RSV)	0	5	0	5	100.0%
Human Rhinovirus (HRV)	0	6	0	6	100.0%
Metapneumovirus (MPVH)	0	5	0	5	100.0%
Influenza A virus (FLU-A)	0	5	0	5	100.0%
Virus influenza B (FLU-B)	0	5	0	5	100,0%

## DISCUSSION

The automated real-time nucleic acid amplification test (Xpert® Xpress SARS-CoV-2) is easy to handle and use. It has demonstrated high diagnostic performance and concordance with the real-time RT-PCR test for the qualitative molecular detection of the virus causing COVID-19 from nasal and pharyngeal swab samples. In addition, it has zero probability of cross-contamination between samples, since all the processes are included in a single cartridge.

Xpert® Xpress SARS-CoV-2 requires minimal infrastructure and biosafety conditions, since it is a fully automated procedure in which the laboratory technician is involved only when loading the sample ^4^. In addition, the short time to obtain results can become an important and vital advantage for patient management. Likewise, this test is highly sensitive, which is supported by its established detection limit of 0.01 plaque forming units (PFU)/ml ^15^.

Different studies have evaluated the performance of this test, finding highly concordant results ^16^^-^^18^. In addition, we did not find cross-reaction, which is in agreement with the findings of Wolters *et al*. ^17^.

On the other hand, we found a discordant result when both tests were evaluated. While the reference method was negative, the evaluated method was positive for the same sample. This could be due to a higher sensitivity of the evaluated method, which could be detecting a lower amount of RNA than the reference method, which is directly related to the detection limit of the test. This fact has already been evidenced in several publications ^11^^,^^19^. In these cases, the result was first reported as indeterminate, then a third test was performed to determine the patient’s final result. As far as possible, this third test should be different from those previously used.

When analyzing the Cycle threshold (CT), the low viral loads found in this study may be due to different factors, such as very early or very late sampling considering the course of the disease, problems during sampling, transport and preservation of the samples, or low levels of viral spread in general. On the other hand, high viral loads may have been obtained in settings where viral spread in the community was active and high. The understanding of viral load levels and the effect this may have on the patient has been very controversial, and different studies in this regard have shown contradictory results ^20^^,^^21^.

Additionally, this test, by having two detection genes, the E gene and the N2 gene, provided significant sensitivity compared to the reference method ^4^. The E gene allows the detection of different SARS (bat coronaviruses), including SARS-CoV-2, so the probable effects of genetic drift can be avoided. Moreover, our results show a wide variation in the viral load obtained (TC values obtained) among the samples that were selected for this study.

Therefore, the evaluated platform offers highly efficient results for the detection of SARS-CoV-2. Its strength lies in the fact that it can be used in a decentralized manner and with fewer resources (human resources, infrastructure, equipment, among others) compared to a traditional real-time RT-PCR. In addition, it works as a multiplatform for the detection of other agents of public health importance from different types of samples. Considering that more than 50 laboratories with this platform have been implemented in Peru by the end of 2023, it could be a potentially useful strategy to establish a significant overall impact in the country, prioritizing those establishments where rapid results are required, such as, for example, hospitals with intensive care in high prevalence environments for the main diseases affecting public health in the country.

The main limitation of the study is that the ideal reference standard was not used, which is the isolation of the virus in cell culture from different samples of a patient; instead, we used the closest standard, such as Rt-PCR, which has been used in multiple studies ^16^^-^^19^. Another limitation is that we did not have data regarding the patient’s disease, such as symptoms, disease duration, or severity. Even so, we consider that these factors affect the evaluated platform and the reference standard equally, so the results are still reliable. On the other hand, it should also be considered that the costs that this platform requires in terms of supplies, equipment and maintenance are still high. Therefore, its prioritization could be a good strategy to address different diseases, accompanied by other technologies that could massively address different diseases.

Finally, we conclude that the diagnostic performance of the Xpert® Xpress SARS-CoV-2 test, in comparison with the reference real-time RT-PCR method for the detection of COVID-19 virus from nasal and pharyngeal swab samples, was highly efficient in terms of sensitivity and specificity. The results obtained for sensitivity and specificity were 100% and 96%, respectively, reaching an excellent correlation according to the results obtained by the kappa coefficient. Likewise, as it is a highly efficient test and easy to implement and use, its decentralized and prioritized use at the points of patient care is recommended.

## References

[1] He F, Deng Y, Li W (2020). Coronavirus disease 2019: What we know?. J Med Virol.

[2] Yuen KS, Ye ZW, Fung SY, Chan CP, Jin DY (2020). SARS-CoV-2 and COVID-19 The most important research questions. Cell Biosci.

[3] Pan American Health Organization (2020). Laboratory Guidelines for the Detection and Diagnosis of COVID-19 Virus Infection.

[4] Xpert Xpress SARS-Cov-2 (2021). Test package insert.

[5] World Health Organization (2014). Xpert MTB/RIF implementation manual: technical and operational 'how-to'; practical considerations.

[6] Haraka F, Kakolwa M, Schumacher SG, Nathavitharana RR, Denkinger CM, Gagneux S (2021). Impact of the diagnostic test Xpert MTB/RIF on patient outcomes for tuberculosis. Cochrane Database Syst Rev.

[7] Nash M, Huddart S, Badar S, Baliga S, Saravu K, Pai M (2018). Performance of the Xpert HIV-1 Viral Load Assay a Systematic Review and Meta-analysis. J Clin Microbiol.

[8] Raja S, Ching J, Xi L, Hughes SJ, Chang R, Wong W (2005). Technology for automated, rapid, and quantitative PCR or reverse transcription-PCR clinical testing. Clin Chem.

[9] Wang W, Xu Y, Gao R, Lu R, Han K, Wu G (2020). Detection of SARS-CoV-2 in Different Types of Clinical Specimens. JAMA.

[10] FIND (2020). COVID-19 NAT Evaluation Protocol Summary.

[11] Zhen W, Smith E, Manji R, Schron D, Berry GJ (2020). Clinical Evaluation of Three Sample-to-Answer Platforms for Detection of SARS-CoV-2. J Clin Microbiol.

[12] Rojas-Serrano N, Lope-Pari P, Huaringa-Nuñez M, Marques Simas PV, Palacios-Salvatierra R, Balbuena-Torres J (2021). Validation and evaluation of RT-PCR real-time in-house test to detection of SARS-CoV-2 using specific RdRp gene and GAPDH endogen control. Rev Peru Med Exp Salud Publica.

[13] Marcos P, Huaringa M, Rojas N, Gutiérrez V, Ruiton S, Gallardo E (2017). Detección de virus influenza A, B y subtipos a (H1N1) pdm09, A (H3N2) por múltiple rt-pcr en muestras clínicas. Rev Peru Med Exp Salud Publica.

[14] Pan American Health Organization (2020). Directrices de laboratorio para la detección y el diagnóstico de la infección por el virus responsable de la COVID-19, 8 de julio del 2020.

[15] Loeffelholz MJ, Alland D, Butler-Wu SM, Pandey U, Perno CF, Nava A (2020). Multicenter Evaluation of the Cepheid Xpert Xpress SARS-CoV-2 Test. J Clin Microbiol.

[16] Vaz SN, Santana DS, Netto EM, Wang WK, Brites C (2021). Validation of the GeneXpert Xpress SARS-CoV-2 PCR assay using saliva as biological specimen. Braz J Infect Dis.

[17] Wolters F, Van de Bovenkamp J, Van den Bosch B, Van den Brink S, Broeders M, Chung NH (2020). Multi-center evaluation of cepheid xpert(r) xpress SARS-CoV-2 point-of-care test during the SARS-CoV-2 pandemic. J Clin Virol.

[18] Moran A, Beavis KG, Matushek SM, Ciaglia C, Francois N, Tesic V (2020). Detection of SARS-CoV-2 by Use of the Cepheid Xpert Xpress SARS-CoV-2 and Roche cobas SARS-CoV-2 Assays. J Clin Microbiol.

[19] Lieberman JA, Pepper G, Naccache SN, Huang ML, Jerome KR, Greninger AL (2020). Comparison of Commercially Available and Laboratory-Developed Assays for In Vitro Detection of SARS-CoV-2 in Clinical Laboratories. J Clin Microbiol.

[20] Lui G, Ling L, Lai CK, Tso EY, Fung KS, Chan V (2020). Viral dynamics of SARS-CoV-2 across a spectrum of disease severity in COVID-19. J Infect.

[21] Liu Y, Yan LM, Wan L, Xiang TX, Le A, Liu JM (2020). Viral dynamics in mild and severe cases of COVID-19. Lancet Infect Dis.

