# Supplementary material for: Diagnostic performance of a point-of-care molecular system for the detection of SARS-CoV-2 in Peru
Source: Rev Peru Med Exp Salud Publica. 2024 Mar 26;41(1):76–82. doi: 10.17843/rpmesp.2024.411.13046 (PMC11149772; doi:10.17843/rpmesp.2024.411.13046)
Supplement: Translation

# Translation: Rendimiento diagnóstico de un sistema molecular de uso en el punto de atención para la detección de SARS-CoV-2 en Perú

**Authors:** Zully M. Puyén, Roger Araujo-Castillo, Jorge Giraldo, Karina Gutierrez, Nancy Rojas-Serrano

**Affiliations:** 1 Instituto Nacional de Salud, Lima, Peru.; 2 Escuela de Medicina, Universidad Peruana de Ciencias Aplicadas, Lima, Perú.

**Keywords:** SARS-CoV-2, COVID-19, Técnicas de Diagnóstico Molecular, PCR, Sensibilidad y Especificidad, Perú

## Abstract

En el presente estudio se estimó el rendimiento diagnóstico de la prueba Xpert®Xpress SARS-CoV-2 en comparación con la RT PCR en tiempo real-protocolo Charité, para la detección de SARS-CoV-2 en pacientes peruanos. Se trató de un diseño de prueba diagnóstica que incluyó 100 muestras de hisopado nasal y faríngeo. Se obtuvo una concordancia global de 98,70% (IC95%: 92,98-99,97), con un coeficiente kappa de 0,97 (IC95%: 0,86-1.00); se estimó una sensibilidad y especificad relativa de 100% y 96,15%, respectivamente. Adicionalmente, el porcentaje del área bajo la curva ROC fue 98,08% en ambos casos y se obtuvo una especificidad analítica del 100% para los diferentes virus respiratorios evaluados. En conclusión, la prueba Xpert®Xpress SARS-CoV-2 a partir de muestras de hisopado nasal y faríngeo fue altamente sensible y específica, así mismo el coeficiente kappa mostró una excelente correlación, al compararla con la prueba de referencia.

## INTRODUCCIÓN

En 2019 se presentaron los primeros casos de la enfermedad causada por el coronavirus (COVID-19) en la provincia de Wuhan, China. Posteriormente, se identificó al virus del SARS-CoV-2 como agente causal ^1^, y a finales de febrero de 2020 fue declarada pandemia por la Organización Mundial de la Salud (OMS) ^2^. Para controlar esta pandemia, uno de los puntos clave fue el desarrollo e implementación de diversas tecnologías para la detección del virus causante de la enfermedad, lo que llevó al desarrollo de pruebas moleculares que ayudaron a una detección rápida y oportuna del SARS-CoV-2 ^3^. Se diseñaron diferentes plataformas con la finalidad de encontrar una alternativa que fuera rápida, de fácil implementación y eficiente. Sin embargo, la mayoría de ellas requieren una infraestructura compleja y personal altamente capacitado, lo cual en muchos casos limita la descentralización de las pruebas moleculares en el Perú.

La plataforma GeneXpert es un sistema cerrado completamente automatizado basado en una PCR en tiempo real, que integra en un solo sistema y automatiza todos los procedimientos de preparación de muestras, extracción y amplificación de ácidos nucleicos, y la detección de las secuencias diana ^4^^,^^5^. Este sistema requiere el uso de cartuchos desechables que incluyen los controles necesarios para validar la corrida de una muestra, así como la detección de los genes diana. En el Perú hasta finales del 2020 se tenían 38 laboratorios implementados con esta plataforma por el Instituto Nacional de Salud (INS) para su utilización en la detección de tuberculosis y/o carga viral de VIH ^6^^,^^7^. Es por ello que una de las estrategias de intervención fue la implementación del cartucho Xpert Xpress SARS-CoV-2 ^4^, el cual fue desarrollado para la detección molecular del virus causante de la COVID-19.

El cartucho Xpert Xpress SARS-CoV-2 detecta específicamente dos genes diana muy importantes, como son el «N2» (nucleocápside proteica) y el «E» (envoltura proteica), aumentando la sensibilidad de la prueba para la detección del SARS-CoV-2 ^8^^,^^9^. El rendimiento obtenido para esta prueba en términos de concordancia fue del 100% de acuerdo con el ensayo realizado utilizando un material de referencia cuantificado de partículas del virus. Asimismo, se estableció el límite analítico de detección (LoD), el cual, según el fabricante, finalmente fue de 0,02 UFP/ml ^4^.

Por todo lo dicho previamente, el objetivo del presente estudio fue evaluar el rendimiento diagnóstico de la prueba de amplificación automatizada del ácido nucleico en tiempo real (Xpert®Xpress SARS-CoV-2) en comparación con la RT PCR en tiempo real-protocolo Charité ^3^, para la detección cualitativa molecular de SARS-CoV-2 (COVID-19), a partir de muestras de hisopado nasal y faríngeo, contenidas en medio de transporte viral universal de pacientes peruanos.

**MENSAJE CLAVE**

Motivación para realizar el estudio: Descripción y evaluación de una plataforma molecular cerrada, de fácil uso y de importancia en el Perú para el manejo de enfermedades de prioridad en salud pública, ahora implementada para la detección de SARS-CoV-2.

Principales hallazgos: Prueba molecular altamente sensible y específica, con una correlación excelente con respecto al referente para detectar SARS-CoV-2.

Implicancias: Puede ser utilizada en los laboratorios que se encuentran en los puntos de atención del paciente para la detección molecular rápida de diferentes agentes infecciosos, incluido el SARS-CoV-2. Se necesita poca experticia y mínima infraestructura para poder implementarla.

## EL ESTUDIO

### Diseño y muestras

El estudio fue de tipo observacional, transversal y el diseño trata de una evaluación de prueba diagnóstica, la cual se llevó a cabo utilizando muestras de hisopado nasal y faríngeo contenidas en medios de transporte universal recolectadas en el año 2020 de pacientes peruanos y que se encontraron almacenadas en el banco de muestras del INS. Estas previamente tuvieron un resultado positivo o negativo mediante RT-PCR en tiempo real para SARS-CoV-2.

Para el cálculo del tamaño muestral se utilizó el programa Epidat 4.2. Considerando un nivel de confianza de 95%, que el RT-PCR clasificó el 50% de muestras como positivas (50 positivas y 50 negativas) ^10^, y asumiendo que la prueba de amplificación automatizada clasificó un número similar como positivas; entonces un tamaño de 100 muestras permite encontrar un coeficiente kappa de Cohen de 0,90 con una precisión de +/- 0,085; una precisión más baja hubiera significado requerir menos muestra. El coeficiente kappa esperado correspondió a una muy buena correlación, cercana al 0,98 reportado por Zheng *et al*. ^11^. Finalmente, las muestras seleccionadas correspondieron a muestras previamente identificadas como positivas (n=52) y negativas (n=50) a SARS-CoV-2 mediante RT-PCR en tiempo real. Dentro de estas 50 muestras negativas, 25 fueron positivas a otros virus con la finalidad de poder evaluar la reacción cruzada.

### Plataformas RT-PCR e instrumentos

*RT-PCR en tiempo real*

La extracción del ARN se realizó siguiendo las especificaciones del fabricante con el kit QIAamp Viral RNA Mini ya sea de manera manual o utilizando el equipo automatizado QIACUBE®. Para la detección del SARS-CoV-2, el extraído fue sometido a la RT PCR en tiempo real-protocolo Charité ^3^ y a la RT-PCR en tiempo real usando cebadores y sondas específicas para los genes RdRP de SARS-CoV-2 y GAPDH de humanos ^12^. Para el caso de la detección de los virus influenza A, influenza B se utilizó la prueba de RT-PCR multiplex de tiempo real en sistema TaqMan (qRT-PCR_Multiplex) ^13^. Finalmente, para la detección del virus sincicial respiratorio, rinovirus humano y metapneumovirus se utilizó una RT-PCR multiplex para otros virus respiratorios diseñado en el INS de Perú. 

*Ensayo de amplificación automatizada del ácido nucleico en tiempo real*

Xpert® Xpress SARS-CoV-2 se realizó de acuerdo con las instrucciones del fabricante para su uso ^4^. Brevemente, se agregaron 300 µL de muestra al cartucho, el cual se cerró y luego se colocó en un equipo GeneXpert IV para realizar la prueba. Los cartuchos incluyen dos controles internos (SPC -control de procesamiento de muestras- y PCC -control de verificación de sonda-) que garantizan el correcto funcionamiento de la prueba ^5^. La detección del SARS-CoV-2 se basa en la identificación de dos genes: «N2» (nucleocápside proteica) y «E» (envoltura proteica), los cuales son utilizados para la interpretación de resultados. Los resultados son generados automáticamente por el sistema GeneXpertTM DX Software versión 6.2. Los resultados son informados de manera cualitativa, y las muestras analizadas son consideradas como positivas cuando se detectan ambos genes N2 y E o cuando se detecta solo N2. Cuando solo se detectó el gen E, la interpretación recomendada por el fabricante es un resultado presunto positivo; sin embargo, en época de pandemia se le consideró como positivo. Esto debido a que, al comienzo de la pandemia, se contaba con pruebas de detección molecular diseñadas para los betacoronavirus, y en época de pandemia el SARS-CoV-2 fue el virus de este género, que circuló más prevalentemente, por lo que en ese momento se recomendó la implementación de la confirmación mediante la detección de un solo marcador genético, teniendo en cuenta que las curvas, así como otros parámetros de aseguramiento de la calidad, fueran óptimos. La PCR del gen E demostró una mejor sensibilidad, por lo que se recomendó priorizar el gen E como el marcador seleccionado ^14^. Sin embargo, luego se comenzaron a hacer pruebas basadas en la proteína S, las cuales fueron más específicas, y es por ello que para las pruebas que salieron posteriormente se incluían adicionalmente este tipo de marcadores para la detección.

### Análisis estadístico

Para el análisis de la información, se utilizó el programa Stata v15.1 (*Stata Corporation, College Station*, Texas, USA). Las variables cualitativas fueron reportadas como frecuencias absolutas y relativas. Las variables numéricas se describieron con mediana y rango intercuartílico (RIC), debido a que el tamaño de muestra pequeño no garantiza una distribución normal; y con rango de valores mínimos y máximos. La concordancia, el rendimiento diagnóstico relativo, el coeficiente kappa de Cohen, con su intervalo de confianza al 95% fueron analizados. Asimismo, se obtuvo la sensibilidad relativa (porcentaje de concordancia positiva) y la especificidad relativa (porcentaje de concordancia negativa) usando el RT-PCR en tiempo real como estándar de referencia imperfecto, esto debido a que, el estándar de referencia ideal es el aislamiento del virus en cultivo celular a partir de diferentes muestras del paciente; sin embargo, por la complejidad del procedimiento este no se suele utilizar por lo que se usó como subrogado la RT PCR en tiempo real. También se calculó el área bajo la curva ROC (Receiver operating characteristic) como un porcentaje con su respectivo intervalo de confianza.

Por otro lado, la especificidad analítica se obtuvo usando solo las pruebas negativas al RT-PCR para SARS-CoV-2 pero positivas para otros virus respiratorios. Todas las evaluaciones se realizaron a un nivel de significancia de 0,05. Se compararon los diferentes parámetros obtenidos, evaluando sus intervalos de confianza cruzados. Si sus intervalos de confianza al 95% no se cruzaron se les consideró estadísticamente diferentes.

### Aspectos éticos

El presente estudio fue aprobado por el comité de ética del INS, con documento N.º RD: 176-2020-OGITT/INS. 

## RESULTADOS

Se incluyeron 102 muestras, 77 procedentes de pacientes sospechosos de COVID-19, recolectadas entre abril y setiembre del 2020; y 25 de muestras prepandemia almacenadas a -80 °C en el banco de muestra del INS. Las primeras fueron utilizadas para calcular el rendimiento relativo de la amplificación automatizada, y las ultimas para evaluar la especificidad analítica de dicha prueba. Del primer grupo, 52 fueron positivas y 25 fueron negativas para SARS-CoV-2 RT-PCR.

El 55,8% (43/77) de las muestras provinieron de personas del sexo masculino. Las mujeres tuvieron una mediana de edad de 40,5 años (RIC: 34-49, mínimo-máximo: 0-60), y los hombres de 47 años (RIC: 38-56, mínimo-máximo: 3-85). Respecto a las muestras procedentes de pacientes prepandemia, 44,0% fueron mujeres (11/25). Sólo hubo una discrepancia cuando se compararon ambas pruebas en pacientes con sospecha clínica de COVID-19, habiendo una muestra negativa por RT-PCR que salió positiva con la amplificación automatizada. Esto arrojó una concordancia global de 98,70% (IC95%: 92,98-99,97), con un coeficiente kappa de 0,97 (IC95%: 0,86-1,00), lo cual corresponde a una correlación excelente. Los números fueron muy similares cuando se excluyeron dos «presuntos positivos» en la prueba de amplificación automatizada, inicialmente considerados como positivos (Tabla 1 y 2).

**Tabla 1:** Comparación de los resultados obtenidos mediante la amplificación automatizada para la detección de SARS-CoV-2, con la prueba RT-PCR en tiempo real.

Criterio de positividad	Resultado de amplificación automatizada	Resultado de RT-PCR en tiempo real		
		Positivo	Negativo	Total
Los presuntos positivos son considerados positivos	Positivo	51	1	52
	Negativo	0	25	25
	Total	51	26	77
Los presuntos positivos son excluidos del análisis	Positivo	49	1	50
	Negativo	0	25	25
	Total	49	26	75

**Tabla 2:** Rendimiento diagnóstico de la amplificación automatizada para la detección de SARS-CoV-2, en comparación con la prueba RT-PCR en tiempo real.

		Detección de SARS-COV-2		
		N ^a^	Resultado (%)	IC95%
Considerando resultados presuntos positivos				
	Concordancia global	76/77	98,70	92,98-99,97
	Coeficiente kappa de Cohen		0,97	0,86-1,00
	Área bajo curva ROC		98,08	94,31-100,00
	Sensibilidad relativa	51/51	100,00	93,02-100,00
	Especificidad relativa	25/26	96,15	80,36-99,90
Excluyendo resultados presuntos positivos				
	Concordancia Global	74/75	98,67	92,79-99,97
	Coeficiente kappa de Cohen		0,97	0,85-1,00
	Área bajo la curva ROC		98,08	94,31-100,00
	Sensibilidad relativa	49/49	100,00	92,75-100,00
	Especificidad relativa	25/26	96,15	80,36-99,90

a Datos evaluados para el análisis específico; IC95%: intervalo de confianza del 95%

ROC: Receiver operating characteristic

La evaluación de rendimiento relativo respecto a RT-PCR en tiempo real para SARS-CoV-2 mostró una sensibilidad relativa de 100%, tanto cuando se incluyeron todas las muestras, como cuando se excluyeron los dos «presuntos positivos» en la prueba de amplificación. La especificidad relativa fue 96,15% y el % del área bajo la curva ROC fue 98,08% en ambos casos (Tabla 1 y 2).

Adicionalmente, en la Tabla 3 se presentan las medianas con sus RIC para los genes RdRP, ORF1a, E, y N del RT-PCR en tiempo real, y para los genes N2, y E de la plataforma de amplificación automatizada utilizada. En general se aprecia un amplio rango de valores para todos los genes evaluados.

**Tabla 3:** Mediana de valores Ct de las pruebas positivas a los diferentes genes utilizados por la RT-PCR en tiempo real y la amplificación automatizada para la detección de SARS-CoV-2.

Plataforma	Gen	N	Mediana	RIC	Min-max
RT-PCR en tiempo real	RDRP	32	22,83	17,71-26,23	10,52-30,55
	ORF	18	21,05	16,82-25,40	13,25-31,81
	GEN_E	26	28,05	23,97-31,97	12,51-38,28
	GEN_N	25	21,62	18,44-27,80	14,67-34,89
Amplificación automatizada	N2_XPERT	50	27,45	21,40-30,70	12,00-39,80
	E_XPERT	49	26,10	18,50-28,50	11,00-44,60

RIC: rango intercuartílico; Min-max: valor mínimo y valor máximo

Finalmente, respecto a la evaluación de especificidad analítica, esta fue de 100% para todos los virus respiratorios empleados, incluyendo el virus sincicial respiratorio (5/5), rinovirus humano (6/6), metapneumovirus (5/5), virus influenza A (5/5), y virus influenza B (5/5) (Tabla 4).

**Tabla 4:** Especificidad analítica de la amplificación automatizada para detección de SARS-CoV-2, relativo al RT-PCR en tiempo real, para diferentes virus respiratorios.

	SARS-CoV-2				Especificidad analítica
	RT-PCR en tiempo real		Amplificación automatizada		
	Positivo	Negativo	Positivo	Negativo	
Virus sincicial respiratorio (VSR)	0	5	0	5	100,0%
Rinovirus humano (RHV)	0	6	0	6	100,0%
Metapneumovirus (MPVH)	0	5	0	5	100,0%
Virus influenza A (FLU-A)	0	5	0	5	100,0%
Virus influenza B (FLU-B)	0	5	0	5	100,0%

## DISCUSIÓN

La prueba de amplificación automatizada del ácido nucleico en tiempo real (Xpert® Xpress SARS-CoV-2) es de fácil manejo y uso. Asimismo, ha demostrado un alto rendimiento diagnóstico y concordancia con la prueba RT-PCR en tiempo real para la detección cualitativa molecular del virus causante de la COVID-19, a partir de muestras de hisopado nasal y faríngeo. Además, presenta nula probabilidad de contaminación cruzada entre muestras, ya que todos los procesos están incluidos dentro de un solo cartucho.

Xpert® Xpress SARS-CoV-2 requiere condiciones de infraestructura y bioseguridad mínimas, ya que se trata de un procedimiento completamente automatizado en donde se involucra al laboratorista solo al momento de cargar la muestra ^4^. Además, el corto tiempo de obtención de resultados puede convertirse en una ventaja importante y vital para el manejo del paciente. Asimismo, esta prueba es de alta sensibilidad, lo que se respalda en su límite de detección establecido, el cual es de 0,01 unidades formadoras de placas (PFU)/ml ^15^.

Diferentes estudios han evaluado el rendimiento de esta prueba, encontrando resultados altamente concordantes ^16^^-^^18^. Además, en el presente estudio no se encontró ninguna reacción cruzada, lo que es concordante con lo encontrado por Wolters *et al*. ^17^.

Por otro lado, se encontró un resultado discordante cuando se evaluaron ambas pruebas. Mientras que el método de referencia resultó negativo, el método evaluado resultó positivo para la misma muestra. Esto podría deberse a una mayor sensibilidad del método evaluado, que podría estar detectando una cantidad de ARN menor que el método de referencia, lo cual está directamente relacionado con el límite de detección de la prueba. Este hecho ya ha sido evidenciado en diferentes publicaciones ^11^^,^^19^. En estos casos, el resultado en primera instancia se reportaba como indeterminado, luego se procedía a realizar una tercera prueba que dirimía el resultado final del paciente. En la medida de lo posible, esta tercera prueba debía ser diferente a las utilizadas previamente.

Al analizar el umbral del ciclo «Cycle threshold» (CT), las cargas virales bajas encontradas en el presente estudio pueden deberse a diferentes factores, como la toma de muestra muy temprana o muy tardía considerando el curso de la enfermedad, problemas en la toma de muestra, transporte y conservación de las muestras, o bajos niveles de diseminación viral en general. Por otro lado, las cargas virales altas pueden haberse obtenido en contextos donde la diseminación viral en la comunidad era activa y alta. La comprensión de los niveles de carga viral y el efecto que ello pueda tener en el paciente ha sido muy controvertida, y diferentes estudios en este sentido han mostrado resultados contradictorios ^20^^,^^21^.

Adicionalmente, esta prueba al tener dos genes de detección, el gen E y el gen N2, proporcionó una sensibilidad importante en comparación con el método de referencia ^4^. El gen E permite la detección de diferentes SARS (coronavirus del murciélago), incluido el SARS-CoV-2, por lo que los probables efectos de la deriva genética pueden ser evitados. Además, en los resultados evidenciados se observa una amplia variación de la carga viral obtenida (valores de CT obtenidos) entre las muestras que fueron seleccionadas para el presente estudio.

Por consiguiente, la plataforma evaluada ofrece resultados altamente eficientes para la detección del SARS-CoV-2. Su fortaleza radica en que se puede utilizar de manera descentralizada y con menores recursos (recursos humanos, infraestructura, equipos, entre otros) en comparación con una RT-PCR en tiempo real tradicional. Además, funciona como una multiplataforma para la detección de otros agentes de importancia en salud pública a partir de diferentes tipos de muestras. Teniendo en cuenta que en el Perú hasta finales del año 2023 se han implementado más de 50 laboratorios con esta plataforma, podría ser una potencial estrategia para establecer un impacto global significativo en el país, priorizando aquellos establecimientos donde se requieran resultados rápidos, como, por ejemplo, en los hospitales que cuentan con cuidados intensivos en entornos de alta prevalencia para las principales enfermedades que afectan la salud pública del país.

La principal limitación del estudio es que no se usó el estándar de referencia ideal, que es el aislamiento del virus en cultivo celular a partir de diferentes muestras del paciente; en cambió se usó el estándar más cercano como es el Rt-PCR, el cual ha sido usado en múltiples publicaciones ^16^^-^^19^. Otra limitación es que no se contó con metadata respecto a la enfermedad del paciente, como síntomas, duración de la enfermedad, o severidad. Aun así consideramos, que estos factores afectan de igual manera a la plataforma evaluada y al estándar de referencia, por lo que los resultados siguen siendo fiables. Por otro lado, también se debe considerar que los costos que esta plataforma, demanda en insumos, equipamiento y mantenimiento aún siguen siendo altos. Por lo tanto, su priorización podría convertirse en una buena estrategia para abordar diferentes enfermedades, acompañadas por otras tecnologías que podrían atender masivamente diferentes patologías.

Finalmente, se concluye que el rendimiento diagnóstico de la prueba Xpert® Xpress SARS-CoV-2, en comparación con el método de referencia RT-PCR en tiempo real para la detección del virus del COVID-19 a partir de muestras de hisopado nasal y faríngeo, fue altamente eficiente en términos de sensibilidad y especificidad. Los resultados obtenidos de sensibilidad y especificidad fueron del 100% y 96%, respectivamente, alcanzando una excelente correlación según los resultados obtenidos por el coeficiente kappa. Asimismo, por ser una prueba altamente eficiente y de fácil implementación y empleo, se recomienda su uso descentralizado y priorizado en los puntos de atención del paciente.
